# Arginine methylation of R81 in Smad6 confines BMP-induced Smad1 signaling

**DOI:** 10.1016/j.jbc.2021.100496

**Published:** 2021-03-03

**Authors:** Jian Wu, Xi Chen, Prerna Sehgal, Tingwei Zhang, Olan Jackson-Weaver, Yongchao Gou, Victoria Bautch, Baruch Frenkel, Hongchen Sun, Jian Xu

**Affiliations:** 1 Center for Craniofacial Molecular Biology, Herman Ostrow School of Dentistry, University of Southern California, Los Angeles, California, USA; 2 College of Stomatology, China Medical University, Shenyang, China; 3 Chongqing Key Laboratory of Oral Diseases and Biomedical Sciences, Chongqing Municipal Key Laboratory of Oral Biomedical Engineering of Higher Education, College of Stomatology, Chongqing Medical University, Chongqing, China; 4 Department of Biology and McAllister Heart Institute, University of Northern Carolina, Chapel Hill, North Carolina, USA; 5 Department of Biochemistry and Molecular Medicine, Keck School of Medicine, University of Southern California, Los Angeles, California, USA; 6 Norris Cancer Center, Keck School of Medicine, University of Southern California, Los Angeles, California, USA

**Keywords:** arginine methylation, PRMT1, BMP, Smad6, Smad1, invasion, osteogenic differentiation, *ALP*, *alkaline phosphatase*, BMP, bone morphogenetic protein, BMPRIAca, constitutively active BMP type IA receptor, co-IP, coimmunoprecipitation, PRMT, protein arginine methyltransferase, PRMT1, protein arginine methyltransferase 1, R74, arginine 74, R81, arginine 81, SAM, S-adenosyl methionine

## Abstract

Bone morphogenetic proteins (BMPs) secreted by a variety of cell types are known to play essential roles in cell differentiation and matrix formation in the bone, cartilage, muscle, blood vessel, and neuronal tissue. BMPs activate intracellular effectors *via* C-terminal phosphorylation of Smad1, Smad5, and Smad9, which relay the signaling by forming a complex with Smad4 and translocate to the nucleus for transcriptional activation. Smad6 inhibits BMP signaling through diverse mechanisms operative at the membrane, cytosolic, and nuclear levels. However, the mechanistic underpinnings of Smad6 functional diversity remain unclear. Here, using a biochemical approach and cell differentiation systems, we report a cytosolic mechanism of action for Smad6 that requires arginine methylation at arginine 81 (R81) and functions through association with Smad1 and interference with the formation of Smad1–Smad4 complexes. By mutating the methylated arginine residue, R81, and by silencing the expression of protein arginine methyltransferase 1, we show that protein arginine methyltransferase 1 catalyzes R81 methylation of Smad6 upon BMP treatment, R81 methylation subsequently facilitates Smad6 interaction with the phosphorylated active Smad1, and R81 methylation facilitates Smad6-mediated interruption of Smad1–Smad4 complex formation and nuclear translocation. Furthermore, Smad6 WT but not the methylation-deficient R81A mutant inhibited BMP-responsive transcription, attenuated BMP-mediated osteogenic differentiation, and antagonized BMP-mediated inhibition of cell invasion. Taken together, our results suggest that R81 methylation plays an essential role in Smad6-mediated inhibition of BMP responses.

Bone morphogenetic proteins (BMPs) are extracellular ligands that carry out their function through the activation of cell surface BMP type I and type II receptors, which subsequently activate the R-Smads, Smad1 and Smad5, *via* C-terminal phosphorylation. The activated R-Smads then form complexes with the Co-Smad, Smad4, and translocate into the nucleus to regulate transcription (1, 2). Among BMP target genes is Smad6, which encodes an inhibitory Smad that restrains the BMP response at multiple levels (3). At the membrane level, Smad6 inhibits BMP-induced Smad1/5 activation through competition for association with the BMP type I receptor (4, 5). At the cytosolic level, Smad6 competes with Smad4 for Smad1/5 binding, therefore disrupting Smad1/5 and Smad4 complex formation (6). At the nuclear level, Smad6 recruits transcription corepressors to suppress BMP-driven transcription (7, 8).

As their name implies, the BMPs control bone development, repair, and regeneration (9). BMPs also regulate a plethora of other biological processes including vascular morphogenesis and inflammation (9, 10, 11, 12, 13, 14). Among roles more recently ascribed to BMP signaling is the inhibition of cell motility. For example, BMP4-induced Smad1 signaling inhibits invasion by hepatic cancer cells (15) and BMP6 signaling represses invasion by breast cancer cells (16).

BMP signaling is regulated by protein methylation on arginine residues (5). The process of protein arginine methylation is catalyzed by a family of nine enzymes termed protein arginine methyltransferases (PRMTs), which methylate histones, signaling mediators, transcriptional regulators, and splicing factors (17, 18). PRMTs have been assigned roles in physiological processes including cell proliferation, survival, and fate determination (18, 19, 20). Among the PRMTs, protein arginine methyltransferase 1 (PRMT1) is responsible for more than 75% of arginine methylation activity in mammalian cells (20) and has been documented to functionally modulate signaling pathways initiated by BMPs, growth factors, cytokines and steroid hormones (5, 21, 22, 23). We have previously reported that PRMT1 methylated Smad6 at arginine 74 (R74) and that this methylation controlled Smad6’s inhibitory function at the cell surface receptor level (5). MS analysis indicated that Smad6 is also methylated on arginine 81 (R81). However, R81-methyl-Smad6 was not detected at the membrane, and its functional significance has not been addressed. In the present study, we show that BMP induces Smad6 methylation at R81 in the cytosol to facilitate its interaction with Smad1 and disruption of Smad1–Smad4 complexes, resulting in repression of Smad1-driven transcription and inhibition of BMP responsiveness.

## Results

### BMP induces PRMT1-dependent R81 methylation of cytosolic Smad6

**Figure 1:**
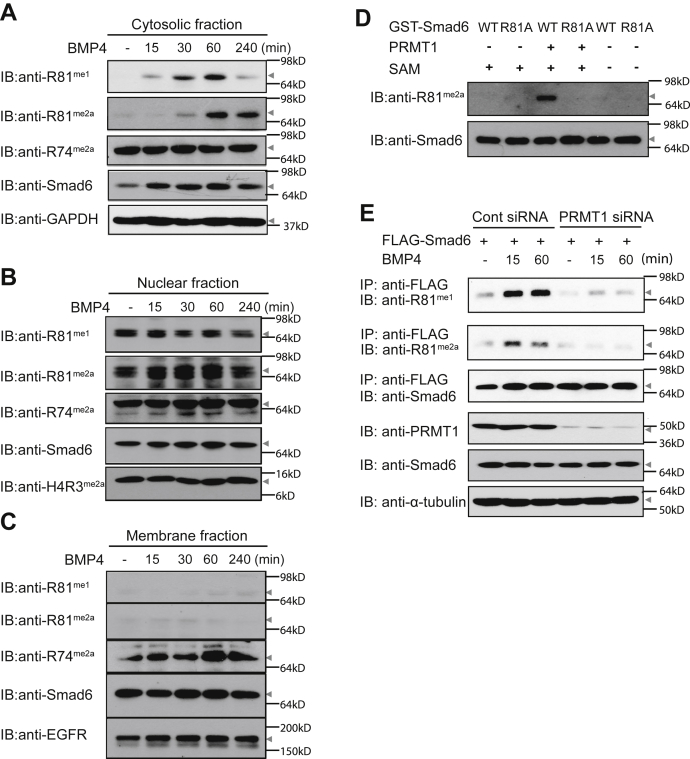
**BMP4 induces Smad6 R81 methylation in a PRMT1-dependent manner.***A*, BMP4 induced monomethylation and dimethylation at Smad6 R81 in the cytosolic fraction, whereas R74 methylation remained steady. HaCaT cells stably expressing Smad6 were treated with BMP4 as indicated, and cytosolic fractions were analyzed by immunoblotting (IB) for R81-methyl-Smad6, R74-methyl Smad6, and total Smad6. GAPDH served as a loading control for the cytosolic fraction. *B*, robust methylation of R74 and R81 sites was observed in the nuclear fraction, although less dependent on BMP4 treatment. Cells were treated as in panel *A*, and nuclear fractions were analyzed as in panel *A*. H4R3me2a served as a loading control for the nuclear fraction. *C*, BMP4-induced R81 methylation is barely detectable at the membrane. Cells were treated as in panel *A*, and membrane fractions were analyzed as in panel *A*. EGFR served as a loading control for the membrane fraction. *D*, PRMT1 methylated Smad6 R81 *in vitro*. Recombinant GST-tagged Smad6 (WT or R81A mutant) and FLAG-tagged PRMT1 were incubated in the methylation reaction buffer with or without SAM followed by IB for Smad6 and R81-methyl-Smad6. *E*, PRMT1 was responsible for Smad6 R81 methylation *in vivo*. HaCaT-FLAG-Smad6 stable cells were incubated with either control siRNA or a mixer of two independent siRNAs targeting PRMT1. Cells were treated with BMP4 for the indicated periods of time and then Smad6 and R81-methyl Smad6 were analyzed by IB. All experiments were independently repeated at least three times. PRMT1, protein arginine methyltransferase 1; R81, arginine 81; SAM, S-adenosyl methionine.

We previously reported that Smad6 is methylated at R74 and R81 (5). At the membrane level, PRMT1-catalyzed Smad6 methylation at R74 controlled BMP signaling activation, whereas R81 methylation was undetectable. To understand the molecular function of Smad6 R81 methylation, we first compared the subcellular localization of R81-methylated Smad6 with R74-methylated Smad6 using specific polyclonal antibodies (5). BMP4-induced methylation of R81 methylation was specifically observed in the cytosolic fraction. Monomethylation of Smad6 R81 was detected after 15 min of BMP4 treatment, followed by asymmetric dimethylation of R81. In contrast, cytosolic Smad6 R74 methylation remained steady (Fig. 1*A*). Robust methylation of both R74 and R81 was observed in the nuclear fraction, although less dependent on BMP signaling (Fig. 1*B*). Consistent with our previous findings (5), BMP4 treatment increased R74 methylation in the membrane fraction, but methyl-R81-Smad6 was barely detectable (Fig. 1*C*).

PRMT1 is the methyltransferase that catalyzes R74 methylation of Smad6 (5). We therefore tested whether PRMT1 also methylated R81 of Smad6. First, using *in vitro* methylation assays, we observed that purified PRMT1 methylated glutathione-*S*-transferase (GST)-tagged recombinant Smad6 at R81 in the presence of methyl donor S-adenosyl methionine (SAM). In control reactions, this methylation did not occur without SAM or when the R81 methylation-deficient mutant R81A was used (Fig. 1*D*). To assess whether PRMT1 is responsible for R81 methylation *in vivo*, we silenced PRMT1 expression using siRNA, which abolished Smad6 R81 methylation in BMP4-treated HaCaT cells (Fig. 1*E*). These findings suggest that BMP4 treatment induces R81 methylation in the cytosolic fraction and that PRMT1 is the major PRMT responsible for Smad6 R81 methylation.

### R81 methylation facilitates Smad6 binding to Smad1

Cytosolic Smad6 has been shown to physically interact with Smad1 and inhibit its nuclear translocation (6). To determine whether R81 methylation regulates Smad6 binding to Smad1, we used the R81 methylation-deficient mutant, Smad6 R81A. In these studies, we avoided using the R81K mutant, which abnormally concentrated in the nucleus, attributable to a cryptic nuclear localization signal (5). The R81A mutant exhibited a normal subcellular distribution (5) and was therefore used in this study.

**Figure 2:**
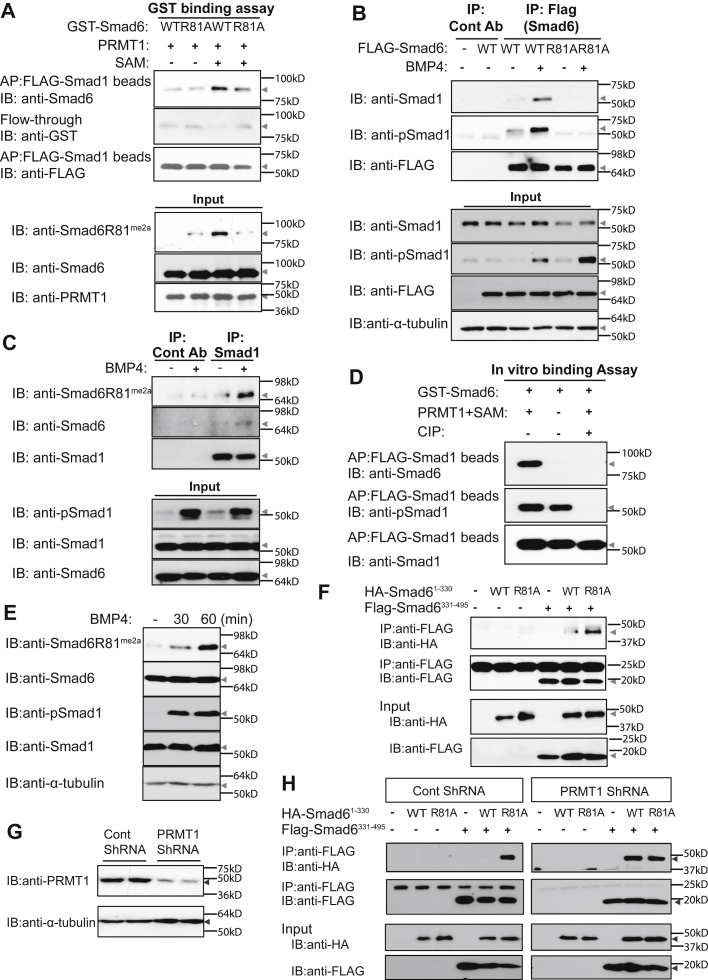
**R81-methylated Smad6 binds to Smad1.***A*, Smad6 R81 methylation was required for binding Smad1 *in vitro*. GST-tagged Smad6 WT or R81A mutant was methylated by recombinant PRMT1 *in vitro* in the presence of SAM and then incubated with FLAG–Smad1–conjugated Sepharose beads. The bound and the flow-through fractions were assessed by immunoblotting (IB). *B*, R81 methylation was required for Smad6 binding to Smad1 *in vivo*. HaCaT cells were transfected with FLAG-tagged Smad6 WT or R81A mutant, treated with BMP4 and subjected to immunoprecipitation (IP) with either anti-FLAG antibody or anti-GFP antibody as control. The presence of Smad1 and pSmad1 in the immunocomplexes was detected by immunoblotting. *C*, endogenous Smad1 interacted with endogenous R81-methylated Smad6. HaCaT cells were treated with BMP4, and endogenous Smad1 was immunoprecipitated with anti-Smad1 antibodies. The coprecipitated Smad6 and Smad1 and the R81 methylation of Smad6 were assessed by immunoblotting. *D*, R81-methylated Smad6 preferred the active phosphorylated Smad1 for binding. GST–Smad6 was methylated by PRMT1 in the presence of SAM and incubated with FLAG-tagged pSmad1-conjugated beads that had been treated with CIP as indicated. The beads were washed and subjected to Western blot (WB) analysis. *E*, BMP4 induced R81 methylation of Smad6 in HaCaT cells. HaCaT cells were subjected to BMP4 treatment followed by WB analysis of R81-methylated Smad6 and Smad1. *F*, the R81A mutant enhanced interaction between Smad6 N-terminal and C-terminal domains. Hemagglutinin (HA)-tagged Smad6^1–330^, either WT or R81A mutant, was coexpressed with FLAG-tagged Smad6^331–495^. The interaction between Smad6^1–330^ and Smad6^331–495^ was assessed by co-IP. *G* and *H*, PRMT1 silencing enhanced the interaction between Smad6 N-terminal and C-terminal domains without further enhancement because of the R81A mutation. Panel *G* shows the efficient silencing of PRMT1 expression. The 293T cells stably expressing control shRNA or shRNA targeting PRMT1 were assessed by immunoblotting. In panel *H*, HA-tagged Smad6^1–330^, either WT or the R81A mutant, was coexpressed with FLAG-tagged Smad6^331–495^ in 293T cells stably expressing control shRNA or shRNA targeting PRMT1. The interaction between Smad6^1–330^ and Smad6^331–495^ was assessed by co-IP. All experiments were independently repeated at least three times. AP, affinity purification; CIP, calf intestinal alkaline phosphatase; PRMT1, protein arginine methyltransferase 1; pSmad1, phosphorylated Smad1; R81, arginine 81; SAM, S-adenosyl methionine.

To examine Smad6 binding to Smad1, we first purified GST–Smad6, either WT or R81A mutant, from *Escherichia coli* and methylated each *in vitro* by incubation with PRMT1 in the presence of SAM. Reactions without SAM provided unmethylated Smad6 as the control. We compared the abilities of R81-methylated and R81-unmethylated WT Smad6 to bind FLAG-tagged Smad1 that had been purified from transfected 293T cells and immobilized on anti-FLAG M2 antibody-conjugated beads. R81-methylated WT Smad6 interacted with Smad1 stronger than either unmethylated WT Smad6 or R81A Smad6 (Fig. 2*A*, first row, third lane *versus* first and fourth lanes). The analysis of Smad6 in the flow-through fractions further supported the results examining the bound Smad6. Specifically, unmethylated WT Smad6 and R81A–Smad6 were present, whereas R81-methylated WT Smad6 was absent in the respective flow-through fraction, indicating retention of the latter on the Smad1-conjugated beads (Fig. 1*A*, second row, third lane *versus* first and fourth lanes). These results provide evidence that Smad6 R81 methylation is required for optimal Smad1 binding.

We further tested the role of R81 methylation in regulating Smad6 interaction with Smad1 in HaCaT cells. Cells were transfected with FLAG–Smad6, either WT or R81A mutant, and treated with BMP4 for 60 min. Smad6 complexes were immunoprecipitated, and the presence of Smad1 in these complexes was assessed by immunoblotting. WT Smad6 interacted with Smad1 in a BMP-dependent manner, whereas Smad6 R81A, which is deficient in R81 methylation, failed to do so (Fig. 2*B*). Furthermore, endogenous Smad6 that complexed with Smad1 in HaCaT cells was R81 methylated (Fig. 2*C*). We noted that Smad6 interaction with Smad1 was enhanced in BMP4-treated cells, in which Smad1 was phosphorylated (Fig. 2C, fourth *versus* Third lane), suggesting that Smad6 might prefer activated Smad1, the C-terminal phosphorylated form (1, 2). We tested this notion by subjecting phosphorylated and unphosphorylated Smad1 to an *in vitro* binding assay similar to that shown in Figure 2*A*. Phosphorylated FLAG–Smad1 was purified from 293T cells coexpressing the BMP type I and type II receptors. To obtain dephosphorylated Smad1, we incubated the purified Smad1 with calf intestinal alkaline phosphatase *in vitro*. The expected levels of Smad1 phosphorylation were confirmed by the WB analysis (Fig. 2*D*, second row). We then tested Smad1 binding to Smad6. As expected, only methylated Smad6 exhibited strong binding to Smad1; remarkably, this binding was observed with phosphorylated Smad1 but not with dephosphorylated Smad1 (Fig. 2*D*). These data suggested that R81-methylated Smad6 prefers phosphorylated Smad1. We further assessed the kinetics of BMP-induced R81 methylation and Smad1 phosphorylation and revealed that BMP-induced Smad1 phosphorylation preceded the peak of Smad6 R81 methylation (Fig. 2*E*).

**Figure 3:**
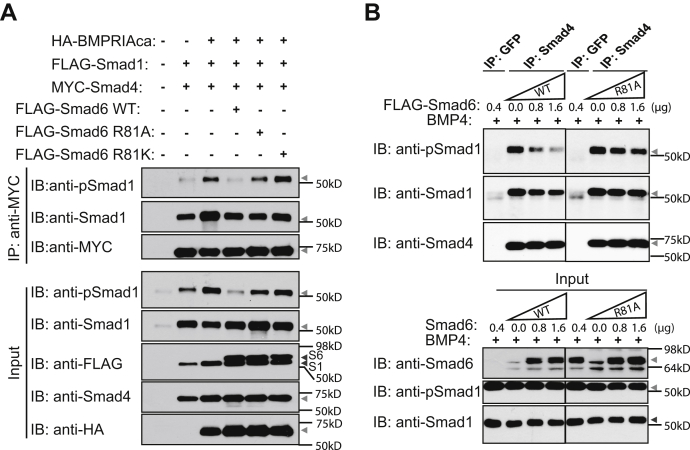
**R81 methylation enables Smad6 to disrupt Smad1–Smad4 complex formation.***A*, the 293T cells were transfected with FLAG-tagged Smad6 WT, the R81A mutant or the R81K mutant, MYC-tagged Smad4, FLAG-tagged Smad1, and HA-tagged constitutively active BMPRIA (BMPRIAca). The interaction between Smad1 and Smad4 was assessed by co-IP with anti-MYC antibody (Smad4) followed by the Western blot analysis of Smad1 and pSmad1. *B*, HaCaT cells were transfected with increasing amounts of FLAG-tagged Smad6 WT or the R81A mutant and treated with BMP4. The interaction between Smad1 and Smad4 was assessed by co-IP with anti-Smad4 antibody followed by Western Blot. Anti-GFP antibody serves as control antibody for IP. All experiments were independently repeated at least three times. co-IP, coimmunoprecipitation; IP, immunoprecipitation; pSmad1, phosphorylated Smad1; R81, arginine 81.

**Figure 4:**
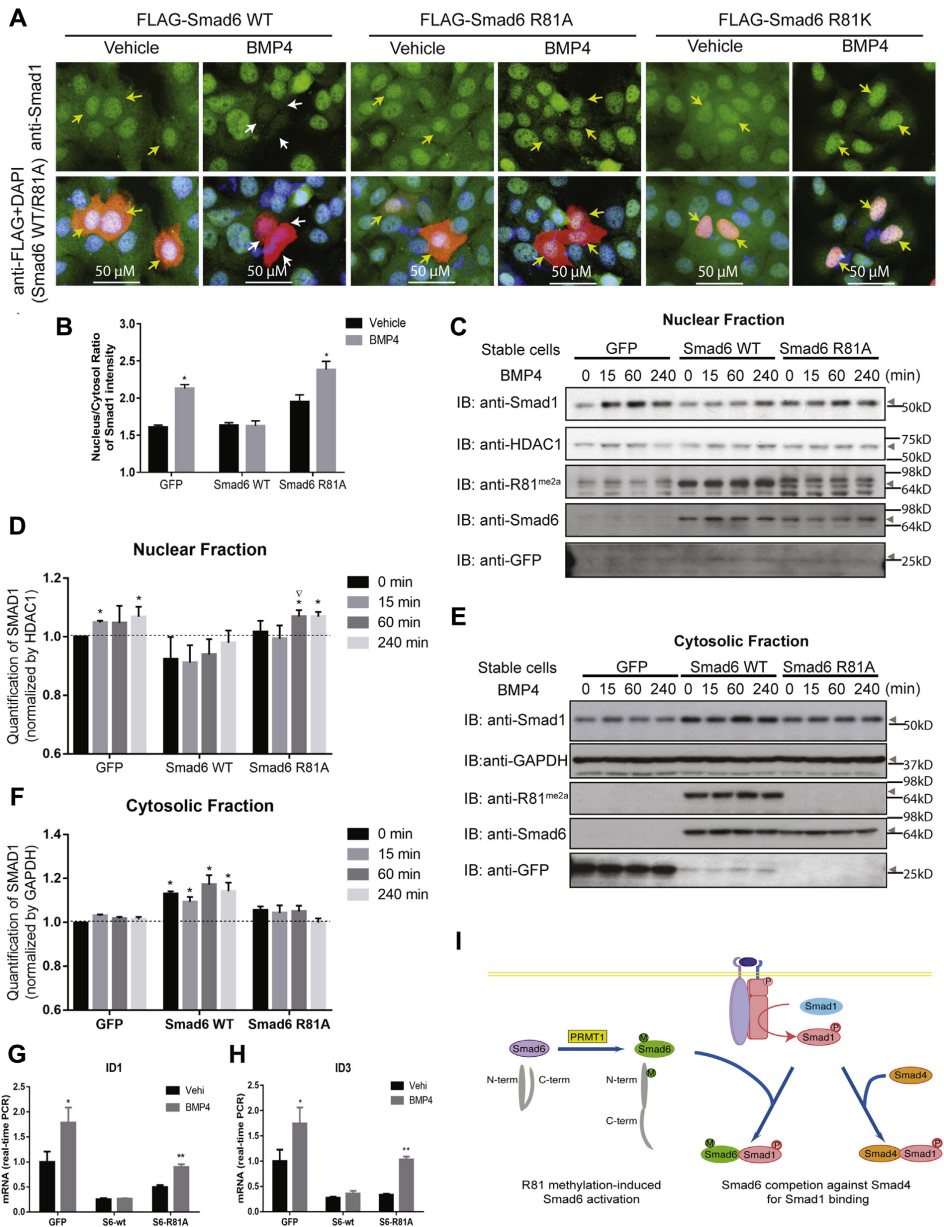
**Smad6 R81 methylation is required for the inhibition of BMP4-induced Smad1 nuclear translocation.***A*, HaCaT cells were transiently transfected with FLAG-tagged Smad6 WT or the R81A mutant or the R81K mutant, treated with BMP4 or vehicle for 1 h and then subjected to immunostaining with anti-Smad1 primary antibodies and Alexa fluor 594–conjugated secondary antibody. Nuclei were imaged based on DAPI staining. The *arrows* indicate transfected cells. Note the weak nuclear Smad1 staining in BMP4-treated cells expressing WT Smad6 (*white arrows*) compared with nontreated cells or cells expressing mutant Smad6 (*yellow arrows*). See Fig. S1*A* for larger fields of view. *B*, HaCaT cells stably expressing Smad6 WT, the R81A mutant, or GFP as control were treated with BMP4 or vehicle for 2 h and subjected to immunostaining with anti-Smad1 antibody. Representative images are shown in Fig. S1*B*. The ratio of nuclear/cytosolic Smad1 was quantified in 100 to 200 cells per group using ImageJ (mean ± SEM). ∗*p* < 0.05, ∗∗*p* < 0.01 *versus* vehicle. *C*–*F*, HaCaT cells stably expressing Smad6 WT, Smad6 R81A, or GFP as control were treated with BMP4 for the indicated periods of time and subjected to cellular fractionation. The cytosolic and nuclear fractions were analyzed by immunoblotting using the indicated antibodies, where HDAC1 and GAPDH serve as loading controls. Immunoblot density was quantified for those in panels *E* and *F* using ImageJ. *G* and *H*, Smad6 WT, but not the R81 mutant, abrogates BMP-responsive gene expression. HaCaT cells stably expressing Smad6 WT, Smad6 R81A, or GFP as control were treated with BMP4 or vehicle for 2 h, and expression of ID1 (*G*) and ID3 (*H*) was measured by RT-qPCR (mean ± SEM). ∗*p* < 0.05, ∗∗*p* < 0.01 *versus* vehicle. All experiments were independently repeated at least three times. *I*, working model: PRMT1 methylates Smad6 at R81, which facilitates Smad6/pSmad1 interaction that competes with Smad1–Smad4 complex formation to inhibit Smad1-mediated BMP signaling. BMP, bone morphogenetic protein; PRMT1, protein arginine methyltransferase 1; R81, arginine 81.

Smad6 interaction with Smad1 occurs *via* its C-terminal MH2 domain (6). Based on previous evidence for interaction between the MH1 and MH2 domains of Smad6 (6, 24), we tested whether R81 methylation in the MH1 domain regulates interaction between the MH1 and MH2 domains of Smad6 to affect Smad6 interaction with Smad1. Smad6 MH1 domain comprising the 330 N-terminal amino acids (Smad6^1–330^) and MH2 domain comprising the C-terminal residues (Smad6^331–495^) were coexpressed in 293T cells, and the interaction between them was interrogated by coimmunoprecipitation (co-IP) assays. The WT Smad6^1–330^ associated with Smad6^331–495^ (Fig. 2*F*, fifth lane), congruent with previous reports (6, 24). Remarkably, we observed much stronger interaction between Smad6^331–495^ and the R81A Smad6^1–330^ mutant (Fig. 2*F*, sixth *versus* Fifth lane). To further confirm that the MH1/MH2 binding difference between the WT and the R81A mutant depended on R81 methylation, and not simply on the arginine residue, we examined the interaction between Smad6^1–330^ and Smad6^331–495^ in PRMT1-depleted 293T cells versus control cells (Fig. 2*G*). Consistent with Figure 2*F*, R81A Smad6^1–330^ bound Smad6^331–495^ much stronger than WT Smad6^1–330^ in control cells. However, WT Smad6^1–330^ and R81A Smad6^1–330^ showed similar binding to Smad6^331–495^ in PRMT1-depleted cells (Fig. 2*H*, right panel, sixth *versus* Fifth lane). These results indicate that Smad6 R81 methylation at the N-terminal MH1 domain reduces the interaction between MH1 and MH2 domains of Smad6. We propose a model whereby R81 methylation of Smad6 decreases the MH1/MH2 intramolecular interaction and facilitates Smad6 association with Smad1.

### R81 methylation enables Smad6 to disrupt Smad1–Smad4 complex formation

We next tested whether the Smad6 R81 methylation–induced interaction with Smad1 inhibited Smad1 binding to the obligatory Co-Smad, Smad4. To this end, we reconstituted a signaling scenario in 293T cells by coexpressing the constitutively active BMP type IA receptor (BMPRIAca) (25), Smad1 and Smad4, along with either WT or mutant Smad6. The BMPRIAca induced Smad1 activation and Smad1–Smad4 complex formation (Fig. 3*A*, first row third lane). Whereas WT Smad6 disrupted Smad1–Smad4 complex formation (Fig. 3*A*, first row fourth lane), the Smad6 R81A mutant did not (Fig. 3*A*, first row fifth lane), suggesting that methylation of Smad6 at R81 is required for its ability to disrupt Smad1–Smad4 complex formation. We also included in this experiment the Smad6 R81K mutant, which mimics WT Smad6 but is unmethylatable at position 81. Smad6 R81K also failed to disrupt Smad1–Smad4 complex formation (Fig. 3*A*, first row sixth lane). These reconstitution experiments altogether strongly suggest a role for Smad6 R81 methylation in disrupting Smad1–Smad4 complex formation.

Finally, we compared WT and mutant Smad6 for their ability to disrupt the formation of endogenous Smad1–Smad4 complexes in HaCaT cells. Smad4-containing complexes were immunoprecipitated from lysates of BMP4-treated cells expressing WT or R81A mutant Smad6, and the presence of phosphorylated Smad1 in the complex was assessed by immunoblotting. Consistent with the reconstituted system (Fig. 3*A*), WT Smad6, but not the R81A mutant dose-dependently inhibited the co-IP of pSmad1 with Smad4 (Fig. 3*B*). Thus, Smad6 R81 methylation is necessary for its ability to disrupt pSmad1–Smad4 complex formation.

### R81-methylated Smad6 inhibits nuclear translocation of Smad1

Because Smad1–Smad4 complex formation is critical for Smad1 nuclear translocation (26), we next tested the role of Smad6 R81 methylation in regulating Smad1 localization. We transiently transfected HaCaT cells with Smad6 WT, the R81A mutant or the R81K mutant, treated them with BMP4, and examined the localization of Smad1 based on immunofluorescence analysis of individual cells. Nuclear Smad1 was hardly detectable in WT Smad6-transfected BMP-treated cells (white arrows in Fig. 4*A* and Fig. S1*A*). In contrast, Smad1 concentrated in nuclei of nontransfected cells in the same field and in cells transfected with the R81A or the R81K mutant Smad6 (yellow arrows in Fig. 4*A* and Fig. S1*A*). We also measured the effect of Smad6 on BMP4-induced Smad1 nuclear localization in HaCaT cell lines stably expressing low levels of Smad6 WT, R81A mutant, or GFP as control. Quantitation of the nuclear and cytoplasmic immunofluorescent signals indicated that BMP4 increased the nuclear-to-cytoplasmic ratio of Smad1 and that this increase was blocked by the presence of Smad6 WT and not R81A (Fig. 4*B* and Fig. S1*B*). The immunofluorescence-based assays were further complemented with a subcellular fractionation approach (Fig. 4, *C*–*F*). We treated the same HaCaT cell lines stably expressing low levels of Smad6WT, R81A mutant, or GFP with BMP4 and analyzed the levels of Smad1 in cytosolic and nuclear fractions. Smad6 WT blocked the BMP4-induced increase in nuclear Smad1, whereas Smad6 R81A failed to do so (Fig. 4, *C* and *D*). Concomitantly, Smad1 accumulated in the cytoplasm of cells expressing WT but not the R81A mutant Smad6 (Fig. 4, *E* and *F*). Consistent with the inhibition of BMP4-induced Smad1 nuclear translocation, WT Smad6, but not the R81A mutant, antagonized BMP-mediated stimulation of the classical Smad1 target genes *ID1* and *ID3* (Fig. 4, *G* and *H*). Thus, R81 methylation of Smad6, which itself is BMP dependent, is required for inhibition of BMP-induced Smad1 nuclear translocation and transcriptional activity.

Taken together, we propose a working model whereby PRMT1-catalyzed Smad6 R81 methylation decreases the interaction between MH1 and MH2 domains and facilitates Smad6 binding to phosphorylated active Smad1, which inhibits Smad1–Smad4 complex formation and Smad1-mediated transcription (Fig. 4*I*).

### R81-methylated Smad6, but not the methylation-deficient mutant, represses osteogenic differentiation

**Figure 5:**
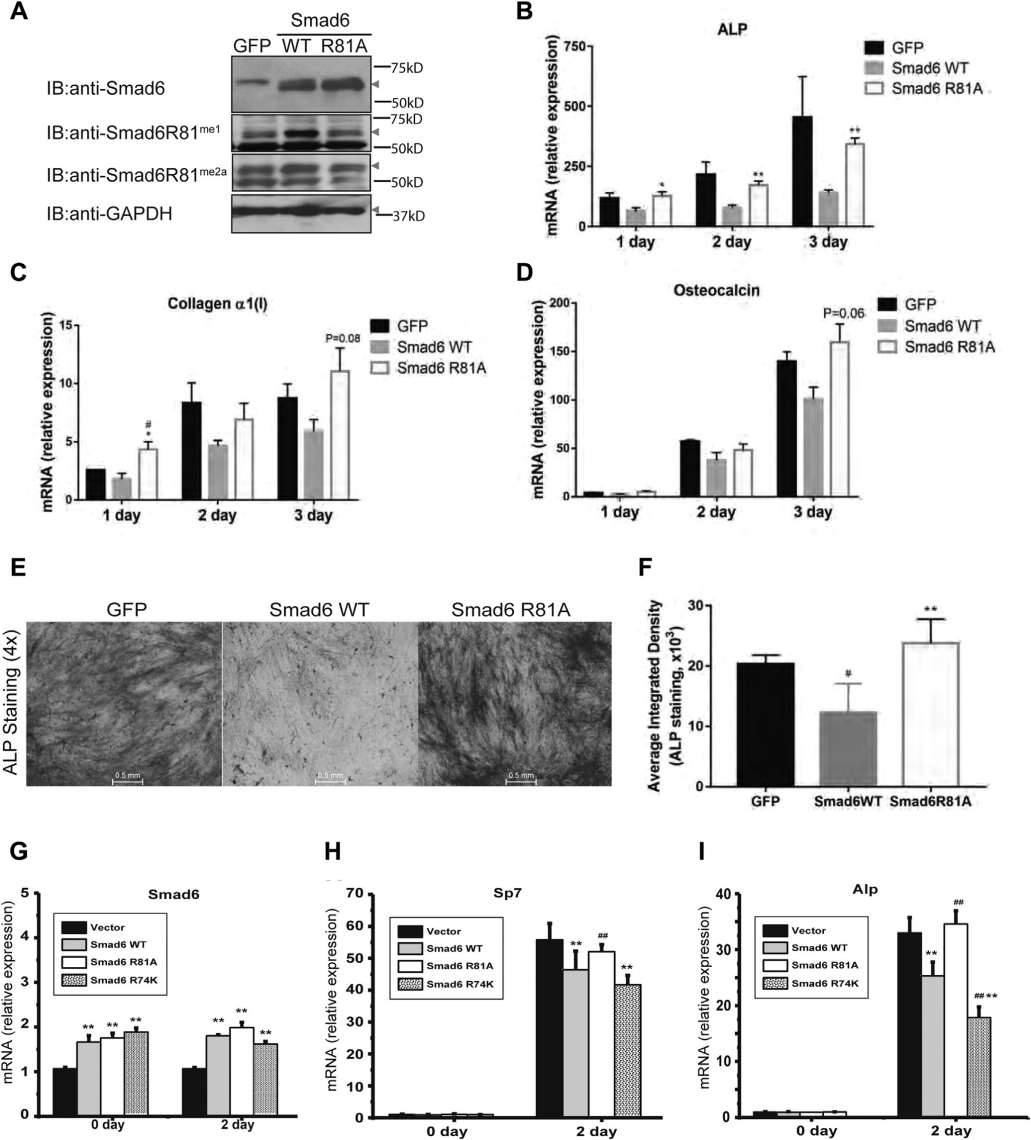
**R81-methylated Smad6 inhibits BMP-induced osteogenic differentiation.** C2C12 cells were transfected with GFP control, WT Smad6, or R81A mutant Smad6 and treated with 200 ng/ml BMP4 to induce osteoblast differentiation. *A*, expression of Smad6 WT and R81A mutant, as well as their R81 methylation, was analyzed by immunoblotting of C2C12 cells after 4 days of BMP4 treatment. GAPDH serves as the loading control. *B*–*D*, expression of *ALP* (*B*), *collagen a1(I)* (*C*), and *Osteocalcin* (*D*) was determined on the indicated days by RT-qPCR. Data were normalized for the respective values measured in the untreated group. *E*, ALP activity in day-5 BMP-treated C2C12 cultures was assessed using histochemical staining. Untreated cultures were negative for ALP. *F*, the average integrated density of ALP staining was measured by using ImageJ (mean ± SEM, n = 3). ∗*p* < 0.05, ∗∗*p* < 0.01 *versus* GFP. *G*, the expression of Smad6 WT and mutants in ST2 cells was analyzed by RT-PCR at day 0 or after 2 days of BMP2 treatment. *H* and *I*, expression of *Sp7* (*H*) and *Alp* (*I*) was determined on the indicated days by RT-qPCR. ^#^*p* < 0.05, ^##^*p* < 0.01 *versus* Smad6 WT. Data were normalized for the respective values measured in the untreated group. All experiments were independently repeated at least three times. *ALP*, *alkaline phosphatase*; BMP, bone morphogenetic protein; R81, arginine 81.

To gain insight into the physiological significance of Smad6 R81 methylation, we first examined its role in the context of osteogenic differentiation. Treatment of myogenic C2C12 cells with osteogenic BMPs induced differentiation toward the osteoblastic phenotype (27). We used this cell culture model to compare the ability of Smad6 WT versus the methylation-deficient R81A mutant to antagonize the effects of BMP4. The WT and R81A mutant Smad6 were expressed at comparable levels and, as expected, only the former was methylated on R81 (Fig. 5*A*). WT Smad6 inhibited the expression of the osteogenic markers *alkaline phosphatase* (*ALP*), *collagen 1α(I)*, and *osteocalcin*, but the R81A mutant did not repress these osteogenic gene markers (Fig. 5, *B*–*D*). Furthermore, histochemical staining for ALP activity showed that Smad6 WT, but not the R81A mutant, inhibited osteogenic differentiation (Fig. 5, *E* and *F*). These data suggest that loss of R81 methylation impairs the inhibitory property of Smad6 in the context of BMP-induced osteogenic differentiation.

Unlike R81, methylation of R74 does not support but, in fact, antagonizes the inhibitory property of Smad6 in BMP-induced signaling (5). At the biochemical level, R81 methylation-deficient mutant slightly reduced R74 methylation (Fig. S2*A*) (28), while R74 methylation-deficient mutant significantly reduced R81 methylation (Fig. S2*A*) (5), indicating that R74 and R81 methylations modulate each other. We further compared the functional significance of R74 and R81 methylation in BMP-induced osteogenic differentiation. ST2 mesenchymal stem cells were treated with BMP2 to induce osteogenic differentiation (29, 30). We compared the ability of Smad6 WT with the methylation-deficient mutants R74K or R81A to antagonize the effects of BMP4. Here, R74K was used because it had fewer mitigating effects on R81 methylation than R74A in previous biochemical assays (5). The WT and mutant Smad6 were expressed at comparable levels (Fig. 5*G*). WT Smad6 inhibited the expression of the osteogenic markers *Osterix*, *ALP*, and *osteocalcin* (Fig. 5, *H* and *I*). Consistent with a role for R81A methylation in Smad6 function, the R81A mutant did not repress these osteogenic markers. In contrast, the R74K mutant exhibited similar or even stronger inhibitory effects (Fig. 5, *H* and *I*). Taken together, these data suggest that R74 and R81 methylations play distinct roles: R74 methylation antagonizes the inhibitory function of Smad6, while R81 methylation is required for the inhibitory property of Smad6 in the context of BMP-induced osteogenic differentiation.

### WT, but not the methylation-deficient R81 mutant Smad6, promotes cell invasion

**Figure 6:**
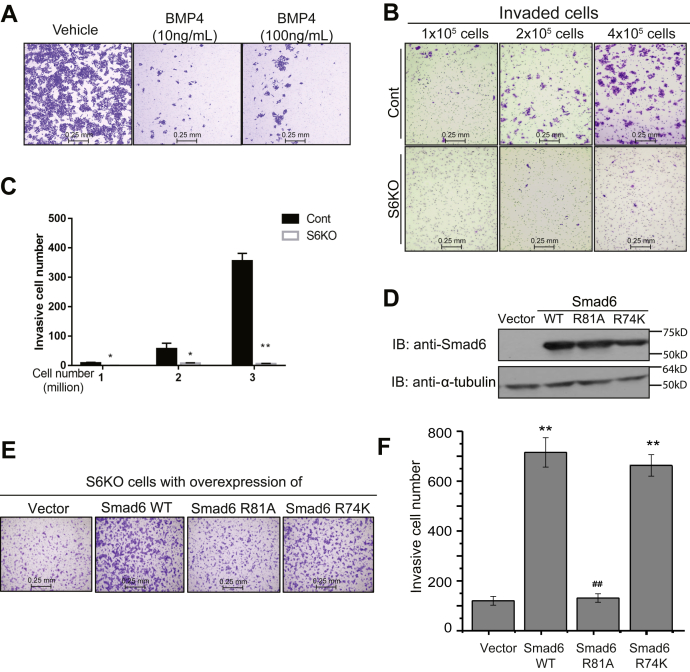
**R81-methylated Smad6 promotes cell invasion.***A*, BMP4 inhibited HaCaT cell invasion. HaCaT cells (2 × 10^5^) were treated with vehicle or BMP4 as indicated and subjected to transwell Matrigel invasion assays. *B* and *C*, Smad6 KO results in dramatically low invasiveness. Increasing numbers of control and Smad6 KO (S6KO) cells were seeded as indicated and subjected to the transwell Matrigel invasion assay. Shown are representative images of invaded cells (*B*) and their numbers (*C*; mean ± SEM; n = 3). *D*–*F*, Smad6 KO cells were transfected with Smad6 WT, the R81A mutant, the R74K mutant or empty vector as the control and subjected to immunoblotting analysis (*D*) and Matrigel invasion assay (*E* and *F*, as in panels *B* and *C*). ∗*p* < 0.05, ∗∗*p* < 0.01 *versus* vector; ^##^*p* < 0.01 *versus* Smad6 WT. All experiments were independently repeated at least three times. R81, arginine 81.

While stimulating osteogenic differentiation, BMP signaling inhibits the invasive phenotype in HaCaT keratinocytes (9) (Fig. 6*A*). In this study, we used Smad6 KO HaCaT cells (28), which are hypersensitive to BMP signaling, marginally less migratory and much less invasive than the parental HaCaT cells (Fig. 6, *B* and *C*, Fig. S2, *B*–*D*). We restored the expression of Smad6 WT in the Smad6 KO cells, or, alternatively, we expressed the Smad6 R81A or R74K mutants and measured cell invasion through Matrigel. Both Smad6 WT and R74K mutant enhanced cell invasiveness, whereas the R81A mutant Smad6 failed to do so (Fig. 6, *D*–*F*). These data suggest that R81 methylation is required while R74 is dispensable for the proinvasive activity of Smad6.

## Discussion

This study provides novel insight into the regulation of BMP signaling. In response to BMP stimuli, Smad6 is methylated at R81, and this facilitates its interaction with Smad1, which in turn disrupts Smad1–Smad4 complex formation, inhibits Smad1 translocation to the nucleus, and represses Smad1-mediated transcription. The Smad6 R81 methylation is therefore expected to play a significant role in regulating physiological responses to BMPs. The present study suggests that it impedes BMP signaling in the contexts of osteogenic differentiation and cell invasion.

The methylation of Smad6 R81 was catalyzed by PRMT1. PRMT1 also methylates Smad6 on the adjacent R74 residue (5, 31). We previously reported that Smad6 R74 methylation was observed at the cell surface rapidly after BMP treatment, preceding Smad1 phosphorylation and activation (5). We demonstrated that R74 methylation dissociated Smad6 from BMP type I receptors as an initiation step of BMP signaling to permit Smad1 activation (5). In the present study, BMP4 induced R81 methylation of Smad6 with a slower kinetics, peaking by 30 to 60 min. Although the Smad6 R74 methylation precedes Smad1 phosphorylation and safeguards signal leakage at the cell surface (5), the Smad6 R81 methylation appears to follow (or parallel) Smad1 phosphorylation and limit the availability of active Smad1 in the cytosol and potentially in the nucleus as well. We also show that R81-methylated Smad6 prefers binding to phosphorylated over unphosphorylated Smad1, indicating that R81-methylated Smad6 preferentially targets active Smad1 to confine BMP signaling. Coordination between Smad6 R74 methylation at the membrane and its R81 methylation at the cytosol is likely required for appropriate control of BMP signaling by Smad6. Loss of Smad6 R81 methylation, or the improper coordination with the R74 methylation, may have undesirable physiological consequences.

We previously demonstrated R81 methylation as a prerequisite for the interaction between Smad6 and myeloid differentiation primary response protein, which facilitated Smurf1 recruitment for myeloid differentiation primary response protein degradation in an oral inflammatory disease, thereby allowing Smad6 in the oral mucosa to inhibit Nuclear Factor kappa B (NF-kB) activation, repress inflammatory chemokine expression, and prevent periodontal tissue degeneration (28). The present study demonstrates that R81 methylation also plays roles in Smad6-mediated inhibition of osteogenic differentiation, as well as Smad6-mediated stimulation of cellular invasiveness, a function recently assigned to Smad6 with implications in multiple types of cancers (16, 32, 33, 34). Unlike R81, methylation of Smad6 on R74 is required for neither the proinvasive nor the antiosteogenic activities of Smad6. R81 methylation may be significant in the context of craniofacial development and cranial suture closure because compromised Smad6 function has been associated with the occurrence of craniosynostosis (35, 36, 37), a congenital condition characterized by premature fusion of bones that form the cranial vault. Finally, because Smad6 modulates BMP responsiveness during vessel sprouting, Smad6 R81 methylation may participate in the angiogenic processes (11, 14).

Our findings indicate that the methylation of Smad6 R74 and that of R81 have distinct biological functions in osteogenic differentiation and cell invasion. However, several important and related questions remain unsolved: how does PRMT1 selectively methylate either R74 or R81? How does R74 and R81 carry out distinct functions in a tissue-specific manner? One potential mechanism is the usage of binding partners, congruent with the notion that protein methylation often alters protein–protein interaction (18, 19, 20). Smad6-binding partners may specifically protect R74 or R81 from methylation by PRMT1. Tissue-specific Smad6-binding partners may facilitate or antagonize downstream activities.

Smad6 modulates various physiological functions, directing and fine-tuning BMP-induced responses in a spatiotemporal-dependent fashion during organ development, tissue repair, and regeneration. Our work demonstrates a significant role for arginine methylation in Smad6-mediated regulation of BMP signaling that enables Smad6 to define BMP signaling kinetics and dynamics. Targeting Smad6 methylation, such as with methyltransferase inhibitors or Smad6 mimicking peptides, may facilitate the development of therapeutic approaches for diseases caused by deregulation of BMP signaling.

## Experimental procedures

### Cell culture

HaCaT, 293T, C2C12, ST2, and their derivative cell lines were cultured in Dulbecco’s modified Eagle’s medium (high glucose, Caisson Laboratories) supplemented with 10% fetal bovine serum bovine serum (10438-026, Gibco). To generate HaCaT stable cells overexpressing Smad6 WT or mutants, FLAG–Smad6WT, FLAG–Smad6R81A, or GFP as control was initially subcloned into the pBabe–puro4 vector using BamHI/XhoI restriction enzymes. The cloned vectors were then cotransfected with pCL–Ampho packaging vector into 293T cells to produce the corresponding retroviruses. Virus suspensions were harvested from cell culture supernatants and then concentrated by 24-h ultracentrifugation at 16,000 rpm. HaCaT cells were infected by the concentrated virus suspensions for 6 h in the presence of 4 μg/ml polybrene. The transduced cells were subjected to 2 μg/ml puromycin selection and single colony expansion. Cell lines expressing similar levels of Smad6 WT or the R81A mutant protein were used in this study. BMP4 for treatment of cells cultures was purchased from HumanZyme and administered at 10 ng/ml unless otherwise stated.

### Plasmids and transfection

Cells were plated 1 day before transfection and allowed to reach ∼60% confluence. The following plasmids were transfected: pcDNA3-FLAG-SMAD6WT, pcDNA3-FLAG-SMAD6R81A, pcDNA3-FLAG-SMADR81K, RK5-HA-SMAD6^1–330^WT, RK5-HA-SMAD6^1–330^R81A, pEGFP-C, pcDNA3-HA-BMPRIA (constitutively active), RK5-Myc-SMAD4, RK5-XF/FLAG-SMAD1. Primers used for PCR cloning are listed in Table S1. For PRMT1 silencing, cells were transfected with siRNAs targeting either PRMT1 or scrambled control siRNA (QIAGEN) using Lipofectamine RNAiMAX (Invitrogen).

### Cell invasion assay

Corning BioCoat Matrigel Invasion Chambers (24-well, Cat# 354480; Thermo Fisher Scientific) were rehydrated with a serum-free medium for 2 h in a tissue culture incubator. Then, 1 to 4 × 10^5^ cells suspended in the serum-free medium were placed in the top chamber and the cell culture medium supplemented with 10% fetal bovine serum was placed in the bottom chamber. After incubation in a tissue culture incubator for 48 h, the noninvading cells together with the Matrigel Matrix were removed from the upper chamber and cells on the lower surface of the membrane were fixed in 4% PFA, stained with 0.05% crystal violet, and imaged with a Keyence BZ-X700 all-in-one fluoresce microscope. Invaded cells were counted in four 20 mm^2^ squares per well.

### ALP staining

Cells were washed with PBS followed by fixation with 10% neutral buffered formalin for 1 min. Cells were washed with 0.05% Tween 20 in DPBS, incubated in BCIP/NBT Liquid Substrate System (Cat# B1911, SIGMA), and washed again with PBS before imaging with a Keyence BZ-X700 all-in-one fluoresce microscope.

### Subcellular fractionation and protein extraction

Cells were trypsinized, washed with ice-cold PBS, collected by centrifugation, and resuspended in ice-cold hypotonic lysis buffer containing 10-mM Tris HCl (pH7.5), 10-mM NaCl, 3-mM MgCl_2_, 0.3% NP-40, 10% glycerol, and protease inhibitors. Cytoplasmic and nuclear fractions were obtained by centrifugation at 800*g* for 10 min at 4 °C. NaCl in the supernatant was adjusted to 140 mM, and the solution, containing the cytoplasmic fraction, was mixed gently and cleared by centrifugation at 20,000*g* for 15 min at 4 °C. The pellet, containing the nuclei, was washed four times with hypotonic lysis buffer and resuspended in the nuclear lysis buffer containing 20-mM Tris HCl (pH 7.5), 150-mM KCl, 3-mM MgCl_2_, 0.3% NP-40, 10% glycerol, and protease inhibitors. After sonication, the nuclear extract was cleared by centrifugation at 20,000*g* for 15 min at 4 °C.

### Western blot analysis

Cells were lysed in the WB lysis buffer containing 1% NP-40, 5% glycerol, Tris-Base, pH 7.5, 250-mM NaCl, EDTA, pH 8.0, and protease inhibitors. The cell suspensions were sonicated for 10 s and centrifuged at 14,000*g* for 15 min. The supernatants were subjected to SDS-PAGE, and the proteins were then transferred to PVDF membranes, which were blocked, and incubated with primary antibodies (overnight at 4 °C) and then with secondary antibodies (1 h at room temperature [RT]). Proteins were visualized using chemiluminescence reagents (GE Healthcare) and X-ray films (Denville Scientific Inc). Rabbit polyclonal antibodies against asymmetric di-methyl R81-Smad6 were generated by New England Peptide LLC as described previously (5); anti-Smad6 (cat# 9519), anti-Smad1/5 (cat# 9516), anti-Smad1 (cat# 6944), and anti-PRMT1 antibodies (cat# 2449) were purchased from Cell Signaling Technology; anti-Smad7 (cat# sc-11392) anti-Smad4 (cat# sc-7966), anti-GFP (cat# sc8334), anti-HDAC1 (cat# sc7872), anti-GST (cat# sc-138) and anti-α-tubulin antibodies (cat# sc-5286) were purchased from Santa Cruz Biotechnology; anti-FLAG antibodies (cat# F3165) and anti-FLAG-M2 affinity agarose (cat# A2220) were purchased from Sigma-Aldrich; Anti-Myc antibodies (cat# 05-724) were purchased from Millipore; and anti-HA (cat# 90515 BioLegend) antibodies (cat# 901515) were from BioLegend; anti-GAPDH (cat# GTX627408) and Anti-BMPRIAca antibodies (cat# GTX113140) were from GeneTex.

### Immunofluorescence

Cells were washed with PBS, fixed in 4% paraformaldehyde for 15 min, and washed again with PBS before 1-h blocking with 10% goat serum containing 0.1% Triton X-100. Incubation with primary antibodies was overnight at 4 °C. Incubation with DAPI and the secondary antibodies, Alexa Fluor 594 goat anti-mouse (Invitrogen, A11005) or Alexa Fluor 488 goat anti-rabbit (Invitrogen), was for 1 h at RT. Cells were then washed and covered with Fluoro-Gel with Tris buffer (Cat: 17985-11, Electron Microscopy Sciences) and visualized using a LEICA DMI3000B/DFC365FX fluorescence microscope.

### co-IP

co-IP was performed essentially as described previously (28). Briefly, cells were lysed in the immunoprecipitation (IP) lysis buffer, and the lysate was cleared by centrifugation at 14,000*g* for 15 min. The IP lysis buffer was identical to the WB lysis buffer, except it contained 150-mM NaCl. After overnight incubation with primary antibodies at 4 °C, protein A or G Sepharose beads (GE Healthcare 17-1279-01 or 17-0618-01) were added for ∼2 h. Beads were gently washed in the IP lysis buffer 3 times and recollected by centrifugation at 6000 rpm for 3 min. Bead-bound proteins were released by boiling in the SDS loading buffer for 10 min before SDS-PAGE. For endogenous protein IP, an extra preclear step was introduced by incubating the lysate with a control rabbit or mouse IgG for 30 min. Beads were removed by centrifugation at 6000 rpm for 5 min before proceeding to incubation with the primary antibodies.

### *In vitro* methylation assay

Approximately 10 to 100 ng GST-Smad6WT or GST-Smad6R81A, purified from *E. coli* culture, was incubated with 0.5 to 1 μg of active PRMT1 (Cat# 14-474; Millipore) in the histone methylation buffer (50-mM Tris HCl, pH 8.0, 10% glycerol, 20-mM KCl, 5-mM MgCl_2_, 1-mM DTT, and 1-mM PMSF) in the presence of methyl donor SAM for 90 min at 30 °C. The reaction was stopped by adding 4X SDS protein loading buffer. Reaction products were subjected to SDS-PAGE, and protein methylation was visualized by WB analysis using anti-R81me2a-Smad6 antibody (NEP Inc).

### *In vitro* GST protein-binding assay

The 293T cells cotransfected with FLAG-Smad1 and BMP receptors RIB and RII were lysed using WB lysis buffer. FLAG–Smad1 was enriched by anti-FLAG M2 affinity agarose beads and incubated with the indicated GST fusion proteins at 4 °C for 4 h, followed by centrifugation at 4000 rpm for 3 min. The supernatant, collected as a flow-through fraction, and the washed beads were subjected to SDS-PAGE. Where indicated, the FLAG–Smad1–conjugated beads were treated by calf intestinal alkaline phosphatase (New England Biolabs) before the binding assay.

### Real-time PCR

Total RNA was extracted with TRIzol (Cat# 15596018; Life Technologies) and iScript Reverse Transcription Supermix (Cat# 1708841; Bio-Rad) was used for cDNA synthesis. Real-time PCR was performed in triplicate using the Universal SYBR Green Supermix (Cat# 172-5271; Bio-Rad) in a Bio-Rad CFX96 Real-Time PCR System. *RPL19* mRNA was used as the internal control. Primers used for RT-qPCR are listed in Table S1.

## Data availability

All the data described are contained within the article.

## Supporting information

This article contains supporting information.

## Conflict of interest

The authors declare that they have no conflicts of interest with the contents of this article.
