# Supplementary material for: Arginine methylation of R81 in Smad6 confines BMP-induced Smad1 signaling
Source: J Biol Chem. 2021 Mar 3;296:100496. doi: 10.1016/j.jbc.2021.100496 (PMC8050389; doi:10.1016/j.jbc.2021.100496)
Supplement: Supplemental Figures S1–S2 and Table S1 [file mmc1.pdf]

Supporting Information

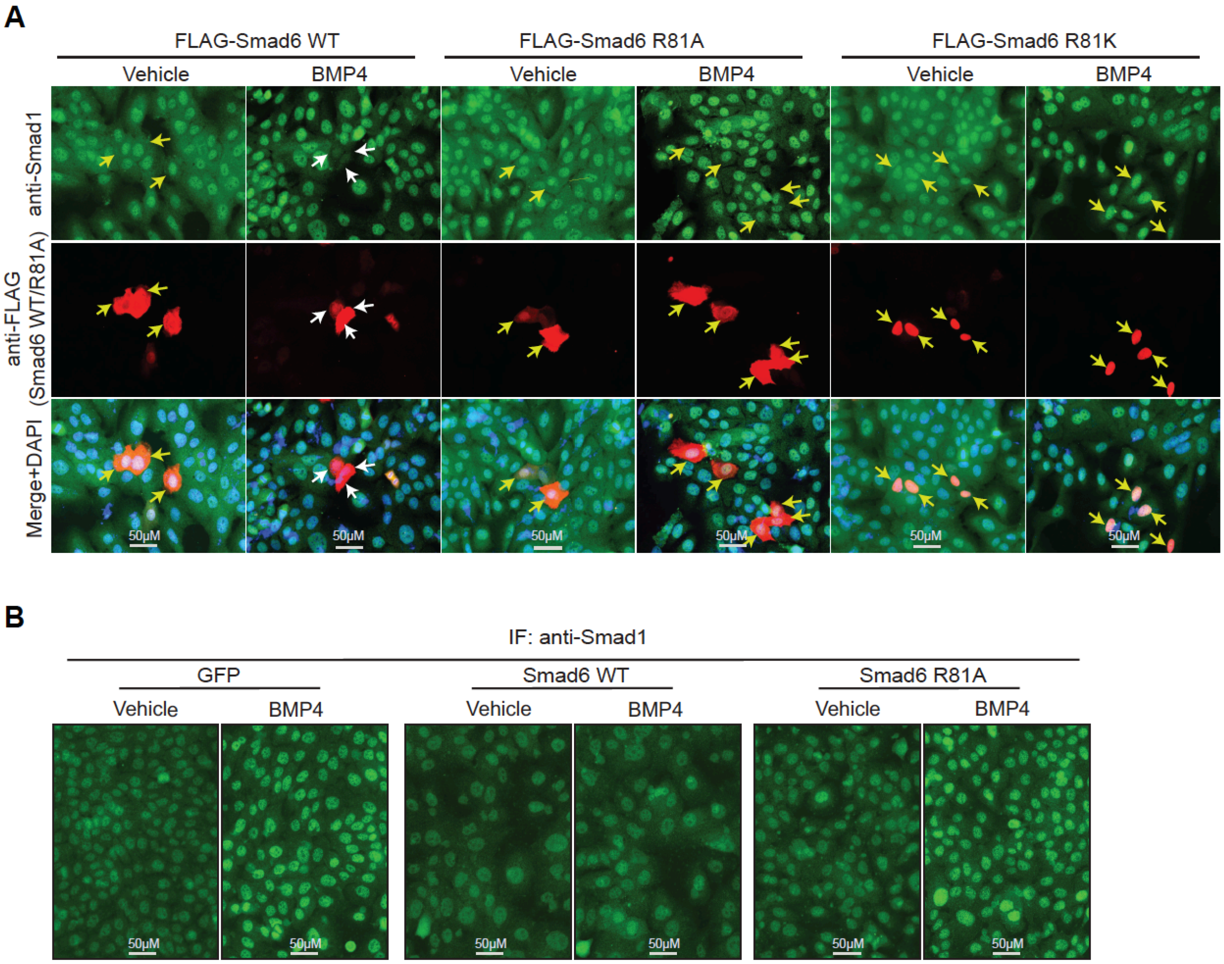

Supplemental Figure S1. (A) HaCaT cells transiently transfected with FLAG-tagged Smad6 WT, the R81A mutant or the R81K mutant were treated with BMP4 or vehicle for 1hr and subjected to immunostaining with anti-Smad1 primary antibodies and Alexa fluor 594-conjugated secondary antibody. Nuclei were imaged based on DAPI staining. Arrows indicate transfected cells. Note the weak nuclear Smad1 staining in BMP4-treated cells expressing WT Smad6 (white arrows) compared to non-treated cells or cells expressing mutant Smad6 (yellow arrows). See Figure 4A for zoom-in views of transfected cells. (B) Representative images of HaCaT cells stably expressing Smad6 WT, the R81A mutant, or GFP as control were treated with BMP4 or vehicle for 2hrs and assessed by immunostaining with anti-Smad1 antibody. The ratio of nuclear/cytosolic Smad1 was quantified in Figure 4B.

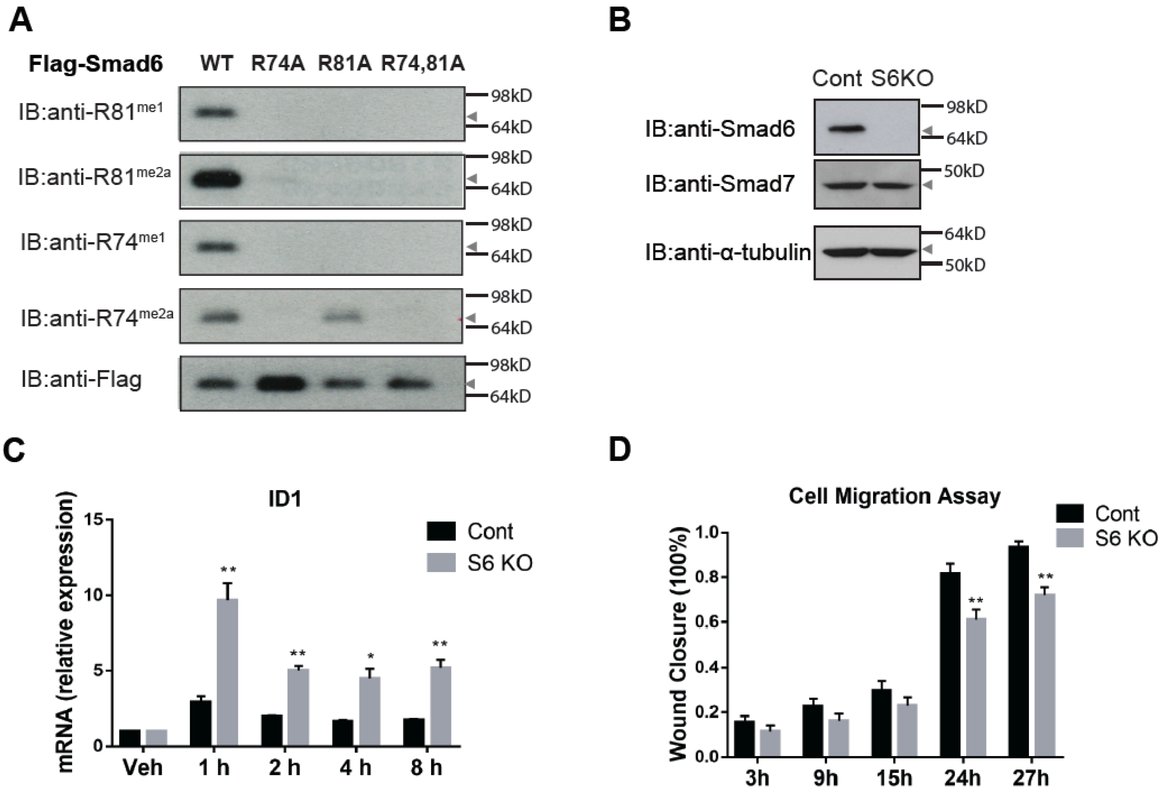

Supplemental Figure S2. (A) FLAG-tagged Smad6 WT or the R74A, R81A or the R74,81A mutant were transiently transfected in 293T cells and subjected to immunoblotting (IB) to assess R81 and R74 methylation using rabbit polyclonal antibodies against mono-methyl-R81-Smad6 (R81me1), asymmetric dimethyl-R81-Smad6 (R81me2), mono-methyl-R74-Smad6 (R74me1) or asymmetric dimethyl-R74-Smad6 (R74me2). IB, immunoblotting. (B-D) Characterization of Smad6 knockout (S6 KO) HaCaT cells. Control or S6 KO HaCaT cells were subjected to IB analysis of Smad6 and Smad7. α-tubulin serves as a loading control. In *C*, cells were treated with BMP4 and *ID1* expression was assessed by RT-PCR. Data are normalized to the vehicle-treated Control group. In *D*, Control or S6 KO HaCaT cells were subjected to wound-healing (scratch) assays. The cell layer was scratched with a pipette tip and the distance between the migratory cell fronts was measured using microscope phase contrast imaging. * $p<0.05$, ** $p<0.01$ *vs.* control.

Supplemental Table 1. Oligonucleotides used in this study

**Oligonucleotides used for RT-qPCR (5'→3')**		
**Name**	**Forward primer**	**Reverse primer**
h*ID1*	CTGCTCTACGACATGAACGG	GAAGGTCCCTGATGTAGTCGAT
h*ID3*	CATTCGTCTACATTCTCGACCTG	TCCTTTTGTCGTTGGAGATGAC
mALP	CCAACTCTTTTGTGCCAGAGA	GGCTACATTGGTGTTGAGCTTTT
m*Collagen1α1*	GCAACAGTCGCTTCACCTACA	CAATGTCCAAGGGAGCCACAT
m*Osteocalcin*	CCGCCTACAAACGCATCTACG	GAGAGAGGACAGGAAGGATCA
mSp7	AAGTTATGATGACGGGTCAGGTACA	AGAAATCTACGAGCAAGGTCTCCAC
